# The Physiognomical Significance of the Teeth

**Published:** 1892-06

**Authors:** George W. Warren

**Affiliations:** Philadelphia


### THE PHYSIOGNOMICAL SIGNIFICANCE OF THE TEETH.

BY GEORGE W. WARREN, D.D.S., PHILADELPHIA.

THE physiognomical function of the face is to depict to the world the true character of the "inner man;" it is the mirror of the emotions; yes, of the heart itself. The faculty of reading the character by the outward natural signs is instinctive in all men, though some have become so skilled by exercising and developing it that they claim to have occult powers, and, with tact and a fertile imagination, do not fail to draw about them many faithful followers. Lavator says, "I am persuaded that faces are as legible as books, and are read in much less time, and are less likely to deceive us; in fact, I cannot recollect that my skill in physiognomy ever deceived me." This man was one of the early students of physiognomy, and about 1800 wrote numerous volumes on the subject. His works are, as he termed them, "fragments;" they are without system; and while they contain a great deal that is good and true, they contain much that is impractical. He has so loaded down the truth with a burden of fantasy that he did not succeed in removing the study from where it had been for centuries,—associated with the so-called occult arts.

The one person who has lifted it from these associations and placed it upon a scientific basis is Mrs. Mary Olmstead Stanton, whose latest work, "A System of Practical and Scientific Physiognomy," is found upon the shelves of our best libraries. It comprises two bulky volumes of as solid and entertaining a piece of literature as often comes from the pen of woman.

In making up the facial features, the jaws and teeth play a very important part. The teeth, standing as they do as guards about the entrance to the digestive tract, tell to the thoughtful student—by their size, form, color, texture, and relative position—not only of the physiological condition of the individual, but of the mental and moral power or weakness which have been created largely by physiological activities. Many suppose that dental prosthesis requires no knowledge other than abstract mechanics or merely manual skill. It is just here, however, in the preparation of artificial dentures that the highest workmanship is called for. Each case involves the necessity of the possession of sufficient knowledge not only of the physiological, but of the physiognomical, condition of the mouth, and skill to restore what is lost. We must first learn to appreciate nature's own work before we can expect to even approximate such perfection. From the similarity of the majority of artificial teeth, there is room in the profession at large for greater study and more artistic work in this branch of our profession. The closest study is needed here to correct the meaningless "horseshoe-style," so prevalent throughout the country. And how often we see even, white teeth where every feature cries out against them. We could hardly find two people with exactly the same facial angle and conformation. The patient's individuality should be well studied before an attempt is made to prepare an artificial denture. The temperament, contour of the face and body, the complexion, age, etc., should be considered, and the artificial denture be constructed in accordance with these physiognomical and other requirements.

Artificial teeth, as manufactured at the depots for the profession, are mere blanks that have to be—or, rather, should be—so shaped and prepared as to harmonize with the facial requirements of each individual patient. Take, for example, a patient of the sanguine temperament. We know that nature gives this temperament teeth that are well proportioned,—abounding in curves, arranged in a full, round arch,—with an articulation that is moderately firm and corresponding generally to the contour of the face and head.

"To individualize the teeth, the cutting-edges of the incisors must be ground to imitate wear by mastication, more or less, according to the age of the patient; the natural occlusion being nearly on end, the front teeth are much worn away. To imitate this waste of tooth-substance gives the operator a fair chance to show his skill and judgment. The cutting-edges of artificial teeth, as received from the depot, are always rounded. This edge should be so ground as to give it a worn appearance. The smallest cut on the edge of each incisor and cuspid will vary the expression greatly, and give character to the teeth according to the slope given and the amount removed, rendering it difficult to recognize them as the same set of teeth."

It is sometimes advantageous to leave out a tooth on one side, say a first bicuspid, and bring the second bicuspid forward about half the width of the tooth removed, representing the loss of a tooth with the space partially closed up. Or a pleasing effect can sometimes be obtained by leaving out the second bicuspid tooth and inserting a gold crown in its place, and very natural effects be made by securing the teeth before they are baked at the depot, and carve and stain them according to the case in hand.

Again, it is in some cases necessary to imitate the irregularities of the natural teeth in order to restore the features; and as Dr. W. R. Hall has said, "This is a very difficult feat to perform, and is seldom successful, as, when attempted, it is usually overdone, and without judgment. Crowding and overlapping gives a confused and bewildering look to the mouth, entirely contrary to the indications of the majority of patients. Irregularities are more numerous in adults of the bilious temperament than any other, and more strongly marked, being in perfect accordance with the rugged characteristics of this type, which is noted for its force, tumultuous passions, and energy of action; but even in this temperament the teeth must be placed and grouped together in such a way as to convey the idea of strength and energy, not a confused jumble."

As another has well said, "If we have not ideal teeth, the probabilities are that there are many other things in feature and complexion which also are far from our highest conception; and the introduction of one or more ideal teeth, where the surroundings are anything but artistic, is no improvement. It creates a discordant note, and destroys the harmony which prevails even in ugliness, and renders that ugliness more evident and more unpleasant. But it requires a high conception of true art to thoroughly appreciate these principles and apply them in practice. It is, therefore, not surprising to find that distinguished dentists are the constant and appreciated friends of men of art and of letters."

